# Lack of Interleukin-6 Affects IFN-γ and TNF-α Production and Early In Vivo Control of *Brucella abortus* Infection

**DOI:** 10.3390/pathogens9121040

**Published:** 2020-12-11

**Authors:** Erika S. Guimarães, Jéssica M. Martins, Marco Túlio R. Gomes, Daiane M. Cerqueira, Sergio C. Oliveira

**Affiliations:** 1 Department of Genetics, Institute of Biological Sciences, Federal University of Minas Gerais, Belo Horizonte 31270-901, Brazil; erikasousaguimaraes@gmail.com; 2 Department of Biochemistry and Immunology, Institute of Biological Sciences, Federal University of Minas Gerais, Belo Horizonte 31270-901, Brazil; jezaid@hotmail.com (J.M.M.); mtrgomes19@gmail.com (M.T.R.G.); daianecerqueira@yahoo.com.br (D.M.C.)

**Keywords:** IL-6, *Brucella abortus*, innate immunity, neutrophils, cell recruitment

## Abstract

Interleukin-6 (IL-6) is a pleiotropic cytokine promptly produced in response to infections, which contributes to host defense through the stimulation of acute phase immune responses. *Brucella abortus* is an intracellular bacterium that causes chronic disease in humans and domestic animals and triggers a robust immune response, characterized by the production of inflammatory cytokines. However, the mechanisms of IL-6-related immune responses in the context of *Brucella* infections are not completely understood. In this report, we describe an increased susceptibility of IL-6 knockout (KO) mice in the early phase of *Brucella* infection. Furthermore, we demonstrate that IL-6 is required for interferon (IFN)-γ and tumor necrosis factor (TNF)-α induction by infected splenocytes, indicating a protective role for IL-6 against *B. abortus* that parallels with Th1 type of immune response. Additionally, IL-6 KO mice exhibited reduced splenomegaly during the early phase of the infection. Corroborating this result, IL-6 KO mice displayed reduced numbers of macrophages, dendritic cells, and neutrophils in the spleen and reduced myeloperoxidase activity in the liver compared to wild-type infected mice. However, we demonstrate that IL-6 is not involved in *B. abortus* intracellular restriction in mouse macrophages. Taken together, our findings demonstrate that IL-6 contributes to host resistance during the early phase of *B. abortus* infection in vivo, and suggest that its protective role maybe partially mediated by proinflammatory immune responses and immune cell recruitment.

## 1. Introduction

Interleukin-6 (IL-6) is an immunoregulatory cytokine with a broad effect on cells of the immune system [1], and high levels of IL-6 are detected during tissue damage, microbial infection, and several other inflammatory conditions [2,3]. Gram-negative bacteria induce increased plasma levels of IL-6 during inflammation, and IL-6 expression has been proposed as a critical activator of protective immunity [4,5,6]. Several studies have shown that IL-6 acts as a powerful inflammatory cytokine that is essential for immune responses in distinct scenarios [7,8,9]. For instance, IL-6 is crucial for inducing an acute-phase inflammatory response against *Listeria monocytogenes* [10] and for the production of interferon-gamma (IFN-γ) during *Chlamydia trachomatis* [11] and *Mycobacterium tuberculosis* infections [6,12]. Furthermore, IL-6 is also required for the production of tumor necrosis factor (TNF-α) during *M. tuberculosis* infection [6].

*Brucella abortus* is a Gram-negative, facultative intracellular coccobacillus, the causative agent of brucellosis, a chronic inflammatory zoonotic disease in humans and livestock. In humans, the disease symptoms include undulant fever and endocarditis arthritis. In animals, it leads to abortion and infertility, resulting in serious economic losses [13,14]. In mice, infection with *B. abortus* provokes rapid activation of the innate immune system, which includes phagocytosis by professional phagocytes, such as macrophages, dendritic cells, and neutrophils. The recognition of bacteria by pattern recognition receptors and cytokine secretion are essential for the restriction of bacterial replication [15]. In particular, the production of Th1-type proinflammatory cytokines, such as IFN-γ and tumor necrosis factor TNF-α, is crucial for the early control of *B. abortus* [16,17,18].

Recruitment of immune cells to the site of infection is a hallmark for many systemic bacterial infections. Previous data showed that IL-6 is closely linked to immune cell influx, and IL-6 deficient mice are unable to induce neutrophilia in the bloodstream following bacterial challenge [19,20]. Furthermore, we have recently shown that neutrophils are crucial for the control of bacteria replication, whereas depletion of these cells renders mice more susceptible to *Brucella* infection [21]. However, the implications of IL-6 in the context of *Brucella* infection are not completely understood, especially due to the lack of studies using IL-6 knockout (KO) mice.

In the present study, we have used IL-6 KO mice to evaluate the role of this cytokine during *B. abortus* infection and provide novel insights on the relationship between IL-6 and innate immune responses in vivo, including the production of other inflammatory cytokines and immune cell recruitment in the context of *B. abortus* infection. Our results indicate that IL-6 plays a protective role in the immune response against *B. abortus* and is crucial for inducing a full inflammatory response. Additionally, we established the importance of IL-6 for inflammatory cell recruitment to the site of the infection, since IL-6 KO infected mice showed a reduced number of macrophages, dendritic cells, and neutrophils in spleens compared to wild-type infected mice. Taken together, these data show the beneficial aspect of IL-6 production and reveal an important role of this cytokine during *B. abortus* infection and pathogenesis.

## 2. Results

### 2.1. IL-6 Is Not Required for In Vitro Control of Brucella Infection

The cytokine IL-6 is largely described for its protective role in immune responses against bacterial infections [10,19,20]. Therefore, to evaluate whether IL-6 affects *Brucella* intracellular replication, we infected wild-type (C57BL/6) and IL-6 KO macrophages with a 100:1 multiplicity of infection (MOI) and quantified the number of *B. abortus* colony forming unit (CFU) in vitro 3 h after infection until 72 h post-infection.

**Figure 1:**
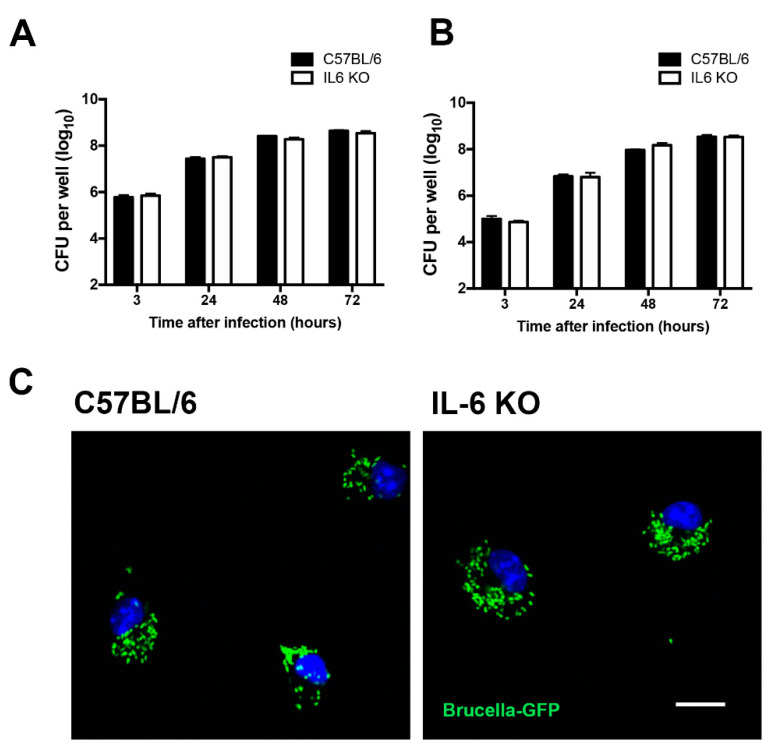
Interleukin (IL)-6 is not involved in *B. abortus* intracellular growth in macrophages. Macrophages derived from wild-type (C57BL/6) and IL-6 KO mice were infected with *B. abortus* for 3, 24, 48, or 72 h. (**A**) Residual CFU in macrophages derived from wild-type and IL-6 KO mice infected with *B. abortus* with MOI = 100:1. (**B**) Residual CFU in macrophages derived from wild-type and IL-6 KO mice infected with *B. abortus* MOI 10:1. (**C**) Macrophages derived from wild-type (C57BL/6) and IL-6 KO mice were infected with *B. abortus*–GFP (MOI = 100:1). Fluorescence microscopy analysis of *B. abortus*–GFP replication 24 h post-infection. Images are representative of all experiments analyzed. GFP-expressing bacteria are shown in green (*Brucella*–GFP) and 4’,6-diamidino-2-phenylindole (DAPI) (DNA) is in blue. Scale bar = 30 mm. Data are representative of three independent experiments.

Wild-type and IL-6 KO infected macrophages showed similar numbers of *B. abortus* at all time intervals analyzed (Figure 1A). To further explore this data, and to evaluate if this result was associated with *Brucella* intracellular infection levels, we performed experiments using a lower MOI (10:1). Nevertheless, lower infection levels also resulted in no difference in CFU numbers between the wild-type and IL-6 KO macrophages at all time intervals studied (Figure 1B), indicating that this event is not associated with the initial *Brucella* numbers. In accordance with these data, confocal images showed similar levels of intracellular *B. abortus*–GFP in wild-type and IL-6 KO macrophages 24 h post-infection (Figure 1C). These findings indicate that IL-6 is not involved in the control of *B. abortus* replication in macrophages.

### 2.2. TNF-α Production by Macrophages Infected with B. abortus Is Partially Dependent on IL-6

**Figure 2:**
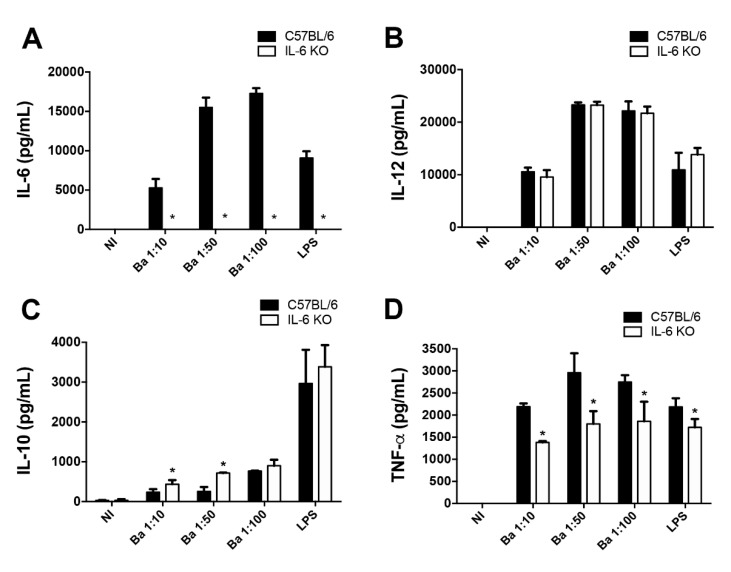
IL-6 modulates proinflammatory cytokine secretion during *B. abortus* infection. Macrophages derived from wild-type (C57BL/6) and IL-6 KO mice were left non-infected (NI) or infected with *B. abortus* (Ba) with MOIs of 1:10, 5:10, or 100:1, as indicated, for 24 h. As a positive control, macrophages were treated with 1 μg/mL of *E*. *coli* LPS for 24 h. The concentration of (**A**) IL-6, (**B**) IL-12, (**C**) IL-10, and (**D**) tumor necrosis factor (TNF)-α by Enzyme-Linked Immunosorbent Assay (ELISA) in the culture supernatants was estimated by ELISA. Data are representative of three independent experiments. * *p* < 0.05 compared to wild-type infected with *B. abortus*, via one-way ANOVA.

The protective role of IL-6 during bacterial infections is regularly mediated through the regulation of other inflammatory cytokines [10,22]. Hence, in order to investigate the role of IL-6 in cytokine production during *B. abortus* infection, macrophages of wild-type and IL-6 KO mice were infected with different *B. abortus* MOIs for 24 h, and cytokine secretion evaluated. Our results show that *B. abortus* infection induced IL-6 secretion in all analyzed conditions (Figure 2A). Furthermore, as expected, the absence of IL-6 (IL-6 KO mice) resulted in abolished IL-6 secretion (Figure 2A) during *B. abortus* infection. As a control, macrophages were treated with lipopolysaccharides (LPS) and, as expected, LPS induced IL-6 secretion in wild-type mice, but not in IL-6 KO (Figure 2A).

In order to investigate IL-6 participation in regulating other immune responses, we evaluated the secretion of the proinflammatory cytokines IL-12 and TNF-α and anti-inflammatory cytokine IL-10 in wild-type and IL-6 KO infected macrophages. IL-6 KO infected macrophages showed similar levels of IL-12 secretion (Figure 2B) in all infection conditions analyzed, compared to wild-type infected macrophages. Additionally, IL-6 KO macrophages stimulated with LPS showed similar IL-12 secretion levels compared to wild-type cells (Figure 2B). Moreover, in the absence of IL-6, IL-10 secretion was slightly enhanced only upon infection with lower bacterial loads (Figure 2C) while IL-6 KO macrophages stimulated with LPS showed no alterations in IL-10 secretion when compared to wild-type cells (Figure 2C). In contrast, the absence of IL-6 resulted in reduced TNF-α secretion compared to wild-type cells (Figure 2D) in all analyzed conditions, including LPS stimulation, and these results were not related to *Brucella* infection levels.

Taken together, these results show that TNF-α production is reduced in macrophages in the absence of IL-6, while IL-10 is mildly altered only with lower bacterial loads, and IL-12 is not affected by IL-6, indicating that IL-6 has a role in regulating TNF-α secretion during *B. abortus* infection.

### 2.3. IL-6 Is Required for Full Resistance During the Early Phase of B. abortus Infection In Vivo

**Figure 3:**
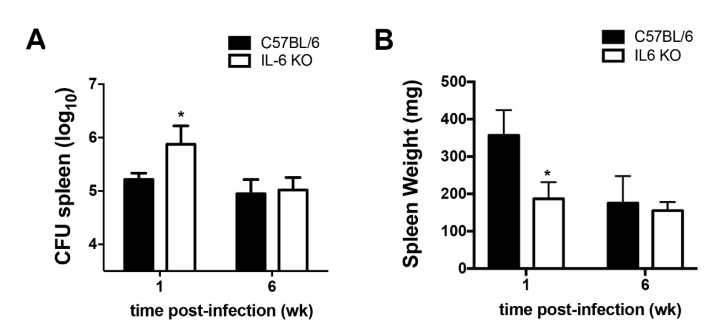
Suppression of IL-6 enhances susceptibility to *B. abortus* infection in mice. Wild-type (C56BL/6) and IL-6 KO mice were infected intraperitoneally with 1 × 10^6^ CFU of *B*. *abortus*. Mice were sacrificed at 1 week and 6 weeks post-infection, spleens were weighed, and diluted spleen homogenates were added to *Brucella* Broth (BB) medium agar plates for CFU determination. (**A**) Residual *B. abortus* CFU in the spleen of wild-type and IL-6 KO mice were determined at 1 week (wk) and 6 weeks post-infection. (**B**) Spleen weight (mg) of wild-type and IL-6 KO infected mice were determined at 1 week and 6 weeks post-infection. Data are representative of three independent experiments. Data are mean ± SD of five mice/group. * *p* < 0.05 compared to wild-type infected with *B. abortus*, via two-way ANOVA.

Our results demonstrated that IL-6 KO macrophages showed reduced TNF-α secretion (Figure 2D). Furthermore, impaired IL-6 function causes enhanced susceptibility of mice to infection by various bacterial pathogens [4,11,23]. Therefore, in order to evaluate the role of IL-6 in host resistance against *Brucella* infection in vivo, we infected wild-type and IL-6 KO mice with *B. abortus*, and CFU counts were analyzed at 1 and 6 weeks post-infection in spleens, an organ that serves as a niche for this bacterium. IL-6 KO mice showed enhanced bacterial numbers at 1 week post-infection compared to wild-type mice, whereas similar bacterial numbers were assessed at 6 weeks post-infection compared to infected wild-type mice (Figure 3A).

Additionally, we evaluated the weight of the spleens of wild-type and IL-6 KO infected mice. Our results demonstrated that IL-6 KO mice showed reduced spleen weight at 1 week post-infection (Figure 3B), and similar spleen weight at 6 weeks post-infection compared to wild-type infected mice (Figure 3B).

Taken together, these results demonstrate that IL-6 is required for early host resistance against *Brucella* infection in vivo, and indicate that IL-6 is required for the splenomegaly induced by *B. abortus*.

### 2.4. Lack of IL-6 Leads to Reduced Type 1 Immune Response to B. abortus

IL-6 is considered an activator of acute phase inflammatory responses [23,24]. Moreover, the production of inflammatory cytokines, such as IFN-γ and TNF-α, is associated with the control of *B. abortus* infection [25,26,27]. Here, we have showed that IL-6 KO mice were more susceptible to *Brucella* infection than wild-type animals during the acute phase of the infection (Figure 3A). Therefore, in order to further analyze the relevance of IL-6 in vivo, we investigated whether the increased susceptibility of IL-6 KO mice during *B. abortus* infection was correlated with altered patterns of cytokine secretion in mice. Splenocytes from wild-type and IL-6 KO mice infected with *B. abortus* for 1 weej and 6 weeks were stimulated with live bacteria, or the controls ConA or LPS, and cytokine secretion was measured.

**Figure 4:**
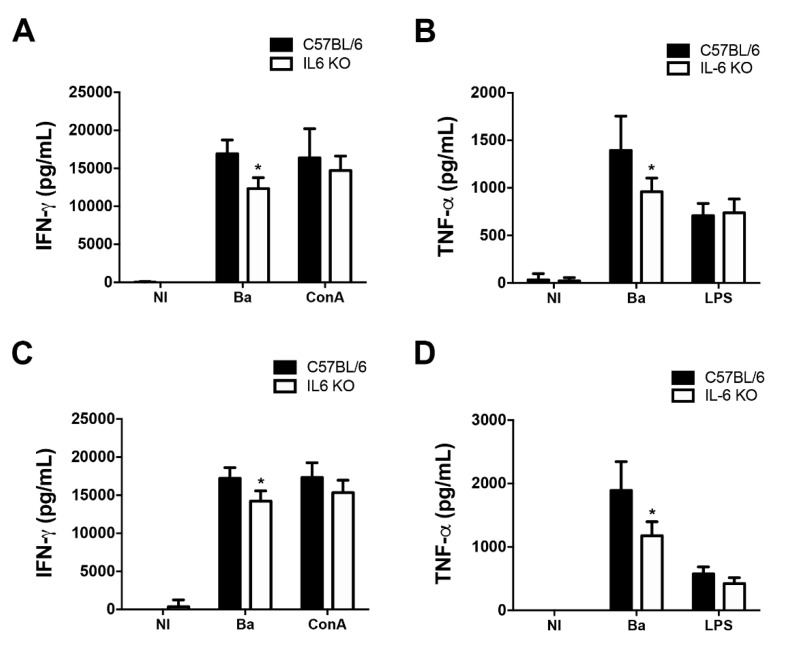
IL-6 is required for interferon (IFN)-γ and TNF-α production by infected splenocytes. Splenocytes of infected mice were stimulated in vitro with *B. abortus* (Ba), 5 μg/mL ConA, 1 μg/mL LPS, or medium (NI) as a negative control. Splenocyte supernatants from 1 week-infected mice were harvested 48 or 72 h after stimulation and measured by ELISA for (**A**) IFN-γ and (**B**) TNF-α. Splenocyte supernatants from 6 week-infected mice were harvested 48 or 72 h after stimulation and measured by ELISA for (**C**) IFN-γ and (**D**) TNF-α. Data are representative of three independent experiments. Data are mean ± SD of five mice/group. * *p* < 0.05 compared to wild-type infected with *B. abortus*, via two-way ANOVA.

IL-6 KO infected mice produced lower levels of the proinflammatory cytokines IFN-γ (Figure 4A,C) and TNF-α (Figure 4B,D), at 1 week and 6 weeks post-infection compared to wild-type infected animals. Furthermore, stimulation with the controls ConA or LPS, respectively, resulted in similar IFN-γ and TNF-α levels, demonstrating the specificity of the response.

Th1 cells are primarily defined by their ability to secret IFN-γ and TNF-α. Furthermore, IFN-γ is known to be the major Th1-inducing cytokine, leading to the polarization of a Th1 phenotype [28,29]. Therefore, these results indicate the ability of IL-6 to regulate, at least partially, immune responses that seem to parallel with type 1 immunity in vivo during *B. abortus* infection.

### 2.5. IL-6 Regulates Innate Immune Cell Recruitment

A typical response to bacterial infections is the immediate recruitment of inflammatory immune cells to the site of infection. Furthermore, it was broadly demonstrated that IL-6 is involved in the recruitment of inflammatory immune cells during infection and inflammation [9,24,29]. Herein, we showed that IL-6 KO mice are more susceptible to early *Brucella* infection in vivo (Figure 3A). Additionally, IL-6 KO mice showed reduced splenomegaly at 1 week post-infection (Figure 3B).

In order to investigate the possible mechanism involved in the increased susceptibility and reduced spleen weight in IL-6 KO mice, we investigated the recruitment of immune cell populations during *B. abortus* infection. Wild-type and IL6-KO mice were infected with *B. abortus*, and 1 week post-infection the number of macrophages, dendritic cells, and neutrophils was evaluated in the spleens by flow cytometry.

**Figure 5:**
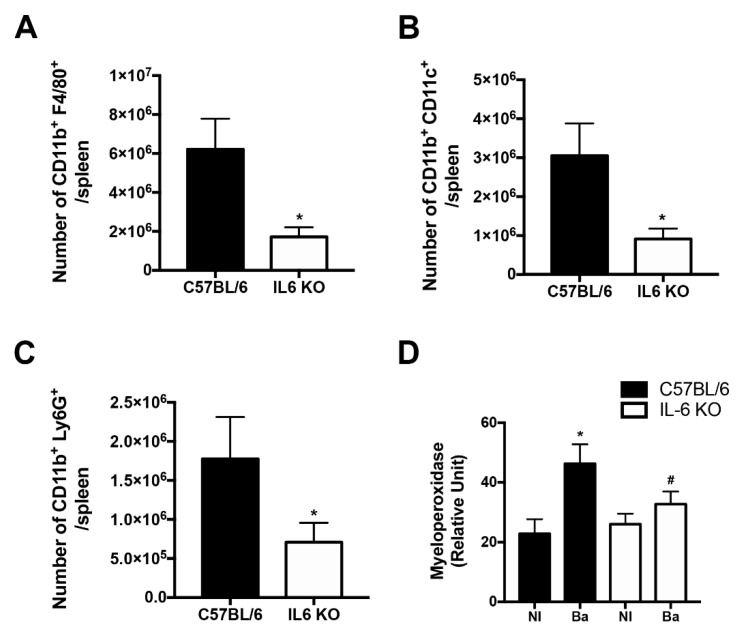
IL-6 regulates immune cell recruitment. Spleen cells from 1 week-infected wild-type (C57BL/6) and IL-6 KO mice were stained ex vivo for flow cytometry analysis. Cells were assessed for (**A**) macrophages (Cd11b^+^F4/80^+^), (**B**) dendritic cells (Cd11b^+^Cd11c^+^), and (**C**) neutrophils (Cd11b^+^Ly6G^+^). Data represent the number of labeled cells (Cd11b^+^F4/80^+^, Cd11b^+^Cd11c^+^, or Cd11b^+^ Ly6G^+^) per number of spleen cells. (**D**) Liver homogenates from wild-type and IL-6 KO non-infected (NI) mice or those infected with *B*. *abortus* (Ba) were submitted to a myeloperoxidase (MPO) assay. Data are mean ± SD of five mice/group. Data are representative of two independent experiments. * *p* < 0.05 compared to wild-type (C57BL/6) non-infected (NI) mice; **^#^** *p* < 0.05 compared to wild-type infected mice (**A**–**C**) via Student’s *t*-test, (**D**) via two-way ANOVA.

IL-6 KO infected mice showed reduced numbers of macrophages (Figure 5A), dendritic cells (Figure 5B), and neutrophils (Figure 5C) in the spleens compared to infected wild-type mice. To further evaluate the potential role of IL-6 in neutrophil recruitment, we submitted liver homogenates from wild-type and IL-6 KO mice infected with *B. abortus* to myeloperoxidase (MPO) assay to indirectly determine neutrophil recruitment. Wild-type infected animals displayed enhanced MPO activity in the livers compared to non-infected animals (Figure 5D), demonstrating that *B. abortus* infection induces neutrophil recruitment. Furthermore, corroborating the reduced neutrophil recruitment in IL-6 KO infected mice spleens compared to wild-type infected mice (Figure 5C), IL-6 KO mice displayed diminished MPO activity (Figure 5D) compared to liver homogenates from wild-type infected mice. Taken together, these findings indicated that IL-6 plays a crucial role in the recruitment of immune cells during *B. abortus* infection and is required for the *Brucella*-induced recruitment of neutrophils.

## 3. Discussion

IL-6 has emerged as a key cytokine during bacterial infections, with complex effects on cells of the immune system and context-dependent proinflammatory described properties [1,30]. Moreover, IL-6 deficiency leads to impaired innate and adaptive immunity to parasitic, viral, and bacterial infections [29,31,32]. Corroborating previous studies [26,33,34], we showed here that *Brucella* induces IL-6 secretion, and it was recently shown that patients with brucellosis exhibit higher IL-6 levels compared to control patients [31]. However, the role of IL-6 during *Brucella* infection is still poorly understood.

In this study, we provided new insights on the relationship between IL-6 and *B. abortus* in vivo infection. We demonstrated that IL-6 is important for the control of *B. abortus* during the early phase of the infection, and demonstrated that the enhanced susceptibility in IL-6 KO mice occurs in conjunction with a decrease in type 1 cytokine secretion and reduced inflammatory cell recruitment.

Previous data demonstrated that macrophages treated with an anti-IL6 monoclonal antibody displayed enhanced susceptibility to *B. abortus* infection in macrophages, suggesting that the induction of IL-6 is required for bacterial killing [32]. Here, we showed that IL-6 is not involved in the control of bacterial replication in macrophages within all analyzed times and infection conditions, indicating that IL-6 is not required for the control of *B. abortus* replication in IL-6 KO macrophages. The discrepancy between these results might be associated with the use of anti-IL6 antibodies in the previous study, since independent studies have shown surprisingly enhanced levels of IL-6 in animals treated with anti-IL-6 antibodies [33,34,35]. In accordance with our data, it was previously reported that IL-6 plays no role in the intracellular growth of *B. abortus* in macrophages [25].

Furthermore, we demonstrated here that IL-6 is critical for the induction of TNF-α secretion in macrophages infected with *B. abortus*, corroborating previous data showing that macrophages treated with IL-6 small interfering RNA (siRNA) and infected with *B. abortus* show reduced TNF-α secretion [32].

Corroborating previous reports demonstrating that IL-6 is often correlated with the susceptibility to infection in several bacterial models [10,19,20], we demonstrated that IL-6 is required for the control of *Brucella* replication in vivo during the early phase of the infection. Accordingly, IL-6 is traditionally considered an activator of acute-phase inflammatory responses [10,24,25].

We demonstrated that the increased bacterial numbers in the spleens of IL-6 KO mice was accompanied by decreased levels of IFN-γ and TNF-α, proinflammatory cytokines critically associated with the control of *B. abortus* infection [25,27]. Indeed, previous studies have demonstrated that IFN-γ KO mice succumb to brucellosis rapidly after infection [16]. Furthermore, IL-6-dependent reduction of IFN-γ was also described for other bacterial infections [6,36]. Additionally, a blockade of TNF-α using specific antibodies likewise increases susceptibility to *B. abortus* [18,37]. Therefore, our findings provide evidence that IL-6 has a protective role in the control of *Brucella* in vivo that is possibly associated with the production of type 1 proinflammatory cytokines. These data support previous findings indicating that IL-6 promotes a Th1 phenotype in inflammatory diseases [36,38,39,40], and also corroborates findings showing that mice treated with an anti-IL-6 antibody and infected with *B. abortus* show reduced IFN-γ, TNF-α, and IL-12 secretion [32].

Here, we demonstrate that despite enhanced bacterial numbers during the early phase of the infection, IL-6 KO mice showed reduced spleen weight at 1 week post-infection. This reduced splenomegaly in IL-6 KO infected mice is most likely related to the impaired recruitment of macrophages, dendritic cells, and neutrophils to the spleens in these mice. Indeed, it was previously shown that injection of recombinant IL-6 into wild-type mice causes an increase in neutrophilia [41]. Furthermore, this result confirms extensive previous studies showing that IL-6 modulates the accumulation of neutrophils during bacterial infections and inflammation [8,19,20,41].

Furthermore, it is well-known that the recruitment of leukocytes is dependent on the specificity of chemokines produced in the inflammatory site. In this regard, interleukin-8 (IL-8) is the most important chemokine for the recruitment of polymorphonuclear cells [42]. It was previously shown that the IL-6-dependent leukocyte recruitment in vivo is linked to the fact that the IL-6– soluble interleukin 6 receptor alpha (sIL-6Ra) complex can activate endothelial cells to secrete IL-8 [43]. Therefore, the IL-6 role in regulating neutrophils recruitment during *B. abortus* infection could be related to the production of other cytokines and chemoattractants in an IL-6-dependent pathway. Additionally, apoptosis has been implicated in the clearance of neutrophils from sites of inflammation [44], and IL-6 was shown to delay neutrophil apoptosis [45]. Therefore, another possible explanation for the reduced neutrophil numbers in IL-6 KO mice is that IL-6 could affect neutrophil apoptosis and removal from the circulation, enabling neutrophil survival during *B. abortus* infection. However, the mechanisms linking IL-6 and neutrophils recruitment during *B. abortus* infection remain to be elucidated.

Additionally, it was recently demonstrated that neutrophils are crucial for the control of *Brucella* replication during the early phase of the infection, since depletion of neutrophils renders wild-type mice more susceptible to *Brucella* infection [21], demonstrating the importance of neutrophils in controlling the early stages of *B. abortus* infection.

In conclusion, our findings revealed that IL-6 enhances type 1 cytokine responses and the recruitment of immune cells during the early stage of infection, two events that are critically associated with the control of *Brucella* infection. Taken together, these data show IL-6 acting as an important regulator of immunity during brucellosis, and imply that IL-6 is a promising therapeutic target during bacterial infections.

## 4. Materials and Methods

### 4.1. Mice

Wild-type C57BL/6 mice were purchased from the Federal University of Minas Gerais, and IL-6 knockout (KO) mice were generated in the C57BL/6 background. Genetically deficient and control mice were maintained at our facilities at the Federal University of Minas Gerais and used at 6–8 weeks of age. Mice were housed in filter-top cages and provided with sterile water and food ad libitum. All animal experiments were preapproved by the Institutional Animal Care and Use Committee of the Federal University of Minas Gerais (CEUA no. 165/2019).

### 4.2. Bacterial Strains

Bacteria used in this study included the *B. abortus* virulent strain S2308 and a variant that constitutively expresses GFP, both obtained from our laboratory collection. Before being used for cell infection, bacteria were grown in *Brucella* broth medium (BD Pharmingen, San Diego, CA, United States) for 3 days at 37 °C under constant agitation.

### 4.3. Cell Culture

Macrophages were derived from the bone marrow of C57BL/6 and IL-6 KO mice and cultured in Dulbecco’s Modified Eagle’s Medium (DMEM) (Life Technologies, Carlsbad, CA, United States) medium, as previously described [46]. Briefly, bone marrow cells were removed from the femurs and tibias of the animals and cultured in 24-well plates (5 × 10^5^ cells per well for cytokine analysis, and 1 × 10^5^ cells per well over a sterile coverslip for microscopy analysis) in DMEM (Life Technologies) containing 10% fetal bovine serum (FBS) (Life Technologies), 1% 4-(2-hydroxyethyl)-1-piperazineethanesulfonic acid (HEPES) buffer solution (Life Technologies), 100 U/mL penicillin (Life Technologies), and 10% L929 cell-conditioned medium, as the source of Macrophage Colony Stimulating Factor (M-CSF) for macrophages at 37 °C in 5% CO_2_.

### 4.4. Infection with B. abortus

Cultured cells were infected in vitro with virulent *B. abortus* strain 2308 with the indicated MOIs (see figure legends) in DMEM (Life Technologies), supplemented with 10% FBS (Life Technologies), for 24 h. In confocal microscopy experiments, macrophages were infected with *B. abortus*–GFP at an MOI of 100:1 DMEM supplemented with 10% FBS (Life Technologies) for 24 h.

### 4.5. Measurement of Brucella CFU in Macrophages

For the measurement of viable intracellular bacteria using CFU, cells infected with the described MOI were washed twice 3 h, 24 h, 48 h, and 72 h post-infection with PBS (Life Technologies), and then lysed for 10 min at room temperature in 1 mL of PBS containing 0.1% Triton X-100 (Sigma-Aldrich, St. Louis, MO, USA) under manual agitation. Lysates were diluted from 10 to 1000 times in PBS and plated on petri dishes containing *Brucella* broth agar. Petri dishes were incubated for 3 days at 37 °C before CFU counting.

### 4.6. Estimation of Intracellular Brucella Numbers by Confocal Microscopy

Macrophages were infected as described above with *Brucella*–GFP (MOI of 100:1) and the bacteria number was assessed in cells infected for 24 h, as previously described [47]. Cells were washed twice with PBS and fixed in 4% paraformaldehyde (pH 7.4) at room temperature for 30 min. After fixation, coverslips were washed three times with PBS. Coverslips were mounted in slides using ProLong Gold with DAPI mounting medium (Invitrogen, Carlsbad, California, USA). Confocal microscopy analysis was performed in a Nikon A1 confocal system. Three coverslips were analyzed per sample and images were taken using a 10× objective for six random areas of each coverslip. Images presented here are representative.

### 4.7. Bacterial Enumeration in B. abortus-Infected Mice

Five mice per group (C57BL/6 and IL-6 KO) were infected intraperitoneally (i.p.) with 1 × 10^6^ virulent *B. abortus* in 100 mL of PBS. After 1 week and 6 weeks post-infection, animals were sacrificed, and spleens were weighed and used to determine the number of bacterial CFU, as previously described [48]. Organs harvested from each animal were macerated in 10 mL of saline (NaCl 0.9%). To determine total bacterial burden, spleens were serially diluted in saline and plated in duplicate on Brucella broth agar. Plates were incubated for 3 d at 37 °C, and CFU number was determined.

### 4.8. Spleen Cell Cultures and Flow Cytometry Analysis

Five mice from each group (C57BL/6 and IL-6 KO) were infected i.p. with 1 × 10^6^ CFU *B*. *abortus* and sacrificed at 1 week post-infection. Spleen cells were harvested treated with an ammonium–chloride–potassium buffer (0.15 M NH_4_Cl, 1.0 mM KHCO_3_, 0.1 mM ethylenediaminetetraacetic acid (EDTA); pH 7.2) to lyse red blood cells. After washing, cells were adjusted to 1 × 10^6^ cells per well in a 96-well plate in RPMI medium (Life Technologies) supplemented with 2 mM L-glutamine (Life Technologies), 25 mM HEPES, 10% heat-inactivated FBS, penicillin G sodium (100 U/mL), and streptomycin sulfate (100 mg/mL). The ex vivo staining of cells was performed as previously described [21]. Briefly, cells were centrifuged at 1500 rpm for 7 min at 4 °C and washed with PBS containing 1% bovine serum albumin (PBS/BSA). Cells were incubated with anti-CD16/CD32 (FcBlock) (1:30 diluted in PBS/BSA) for 20 min at 4 °C. The cells were then centrifuged and washed in PBS/BSA and incubated for 20 min at 4 °C with a mixture of the following antibodies: rat IgG2a anti-murine F4/80 conjugated to biotin (clone BM8; 1:200); rat IgG2b anti-murine CD11b conjugated to APC-Cy7 (clone M1/70; 1:200); hamster IgG1 anti-murine CD11c conjugated to FITC (clone HL3; 1:200); rat IgG2a anti-murine Ly-6G conjugated to PE (clone 1A8; 1:200) and rat IgG2a anti-murine CD62L conjugated to APC (clone MEL-14; 1:400). All antibodies were obtained from BD Pharmingen. Cells were centrifuged and washed again with PBS/BSA and incubated with streptavidin conjugated to PerCP Cy5.5 (1:30) for 20 min at 4 °C. Finally, cells were washed three times, suspended in PBS buffer, and evaluated using Attune Acoustic Focusing equipment (Life Technologies). Results were analyzed using FloWJo software (Tree Star, Ashland, OR, USA) and represent the number of labeled cells (macrophages: Cd11b^+^ F4/80^+^; dendritic cells: Cd11b^+^ Cd11c^+^; or neutrophils: Cd11b^+^ Ly6G^+^) per number of spleen cells.

### 4.9. Cytokine Measurements

For cytokine determination, macrophages were seeded at a density of 5 × 10^5^ cells per well in 24-well plates and infected with the described *B*. *abortus* MOI, as described above, for 24 h. As a positive control, cells were primed with 1 μg/mL of *E*. *coli* LPS (Sigma) for 24 h. Then, supernatants from cell culture were harvested and assayed for the production of murine IL-6, IL-12, IL-10, and TNF-α by ELISA (R&D Systems, Minneapolis, MN, USA), according to the manufacturer’s instructions. For the in vivo experiments, spleen cells were harvested from five infected mice from each group (C57BL/6 and IL-6 KO) at 1 week and 6 weeks post-infection, as described above. To measure cytokine concentration by ELISA, splenocytes were stimulated with *B. abortus* at an MOI of 100:1, 1 μg/mL *Escherichia coli* LPS (Sigma), or 5 μg/mL concanavalin A (ConA) (Sigma). Unstimulated cells were used as the negative control (NI). Spleen cells were incubated at 37 °C in 5% CO_2_, and culture supernatants were harvested 48 or 72 h after stimulation to measure TNF-α or IFN-γ, respectively.

### 4.10. Myeloperoxidase Activity

Five mice per group (C57BL/6 and IL-6 KO) were infected i.p. with 1 × 10^6^ virulent *B. abortus* in 100 mL of PBS. At 1 week post-infection, animals were sacrificed and their livers homogenized using a tissue homogenizer (T10 Basic ULTRA-TURRAX, IKA, Königswinter, Germany). Liver homogenates used to determine myeloperoxidase (MPO) activity, as previously described [49], as an indirect measure of neutrophil numbers [50]. In short, the livers were homogenized in 50 mM HEPES buffer (pH 8.0) and centrifuged, and the pellets were rehomogenized in H_2_O/0.5% cetyltrimethylammonium chloride (CTAC) (Merck, Darmstadt, Germany). After centrifugation, supernatants were diluted in 10 mM citrate buffer, pH 5.0/0.22% CTAC, and substrate solution (3 mM 3,5,5-tetramethylbenzidine dihydrocloride (TMB) (Sigma), 120 M resorcinol (Merck), and 2.2 mM H_2_O_2_ in distilled water) was added. These reaction mixtures were incubated for 30 min at 37 °C and stopped by the addition of H_2_SO_4_. Optical density (OD) at 450 nM was determined.

### 4.11. Statistical Analysis

The results of this study were analyzed using two-way ANOVA, one-way ANOVA, or Student’s *t*-test, as specified in each figure legend, with GraphPad Prism 5 computer software (GraphPad Software, San Diego, CA, USA). Differences were considered statistically significant at a *p* value < 0.05.
